# Real-World Outcomes of Ruxolitinib as Salvage Therapy in Steroid-Refractory Acute and Chronic Graft-Versus-Host Disease: A Multicenter Retrospective Observational Study from Turkey

**DOI:** 10.3390/jcm15052088

**Published:** 2026-03-09

**Authors:** Mehmet Bakırtaş, İlhami Berber, İpek Yönal Hindilerden, Mehmet Sinan Dal, Şebnem İzmir Güner, Ayşe Uysal, Ömer Ekinci, Burcu Aslan Candır, Bülent Eser, Seval Akpınar, Soykan Biçim, Tuğçe Nur Yiğenoğlu, Turgay Ulaş, Burhan Turgut, Mehmet Ali Erkurt, Fevzi Altuntaş

**Affiliations:** 1 Department of Hematology, Tekirdağ Dr. İsmail Fehmi Cumalıoğlu City Hospital, Tekirdağ 59030, Turkey; 2 Department of Hematology, Faculty of Medicine, İnonü University, Malatya 44280, Turkey; drilhamiberber@hotmail.com (İ.B.);; 3 Division of Hematology, Department of Internal Medicine, İstanbul University İstanbul Medical Faculty, İstanbul 34100, Turkey; ipekyonal@hotmail.com; 4 Department of Hematology & Apheresis Unit, Ankara Oncology Training and Research Hospital, University of Health Sciences, Ankara 06200, Turkey; dr.sinandal@gmail.com (M.S.D.);; 5 Department of Adult Bone Marrow Transplantation Unit & Hematology, Hisar Intercontinental Hospital, İstanbul 34100, Turkey; sebnemizmirguner@gmail.com; 6 Division of Hematology, Department of Internal Medicine, İstanbul Gelişim University, İstanbul 34100, Turkey; 7 Hematology Department, School of Medicine, Fırat University, Elazığ 23000, Turkey; 8 Hematology and Bone Marrow Transplantation Unit, Şişli Kolan International Hospital Istanbul, İstanbul 34100, Turkey; 9 Department of Hematology, Adıyaman Training and Research Hospital, Adıyaman 02200, Turkey; drburcuaslancandir@gmail.com; 10 Department of Hematology, Medikal Park Antalya Hospital, Antalya 07160, Turkey; 11 Department of Hematology, Faculty of Medicine, Tekirdağ Namık Kemal University, Tekirdağ 59030, Turkey; 12 Malatya Education and Research Hospital, University of Health Sciences, Malatya 44280, Turkey; 13 Division of Hematology, Department of Internal Medicine, School of Medicine, Ankara Yıldırım Beyazıt University, Ankara 06200, Turkey

**Keywords:** hematopoietic stem cell transplantation, graft-versus-host disease, Janus Kinase Inhibitor, ruxolitinib, survival analysis, Adverse Drug Event

## Abstract

**Introduction & Objective:** Graft-versus-host disease (GVHD) is a major complication of allogeneic hematopoietic stem cell transplantation (allo-HSCT), with limited treatment options for steroid-resistant cases. Ruxolitinib, a JAK1/2 inhibitor, has shown promise in treating steroid-resistant acute (aGVHD), chronic (cGVHD), and overlap GVHD (oGVHD), but real-world data remain limited. This study evaluated the real-world efficacy and safety of ruxolitinib in allo-HSCT patients with steroid-resistant GVHD. **Materials & Methods:** This retrospective, multicenter study included adult patients treated with ruxolitinib for Grade II or higher aGVHD or moderate-to-severe cGVHD at nine centers in Turkey (2017–2024). Clinical characteristics, treatment responses, and adverse events were recorded. Primary outcomes were overall response rate (ORR) and overall survival (OS). **Results:** Among 80 patients (mean age: 39.3 ± 13.3 years; 60 males), 39 had aGVHD, 68 cGVHD, and 15 oGVHD. The ORR was 72 of 80 patients (90.0%) (complete response: 37 of 80 [46.3%], partial response: 35 of 80 [43.8%]). The 1-year and 2-year OS rates were 91.3% and 82.5%. Severe cGVHD (*p* < 0.001) and lack of response to ruxolitinib (*p* = 0.018) were associated with reduced OS. Adverse events included infections in 40 of 80 patients (50.0%), cytopenias in 23 of 80 (28.7%), and cytomegalovirus reactivation in 20 of 80 (25.0%). **Conclusion:** In this retrospective multicenter cohort, ruxolitinib was associated with high response rates in steroid-refractory GVHD, while disease severity remained a key determinant of survival, and findings should be interpreted as exploratory.

## 1. Introduction

Graft-versus-host disease (GVHD) is one of the most devastating and life-threatening complications of allogeneic hematopoietic stem cell transplantation (allo-HSCT) performed for a number of benign and malignant hematological disorders [1,2,3,4,5]. There are limited treatment options that can be used to treat GVHD once it occurs [3]. Although high-dose steroids are used as the first-line treatment modality for GVHD, their efficacy is not more than 50% [4]. Therefore, various second-line therapies have gained popularity in recent years, such as cyclosporine A, sirolimus, tacrolimus, mycophenolate mofetil, pentostatin, infliximab, daclizumab, alemtuzumab, mesenchymal stroma cells, and ruxolitinib [3,6]. However, there is not yet an established standard salvage therapy for steroid-resistant GVHD after allo-HSCT [5,6,7,8]. Ruxolitinib, a selective oral Janus-associated kinase (JAK)1/2 inhibitor, was initially approved for treating myelofibrosis [1,9,10]. The primary mechanisms of action of ruxolitinib are reduction in pro-inflammatory cytokine release and inhibition of allogeneic T-cell proliferation [1]. Considering these pathophysiological mechanisms, ruxolitinib has been used to control and prevent transplant-related complications, most notably GVHD, and emerged as a potent modulator of GVHD subtypes, i.e., acute (aGVHD), chronic GVHD (cGVHD) and overlap GVHD (oGVHD) [1,2,4,11,12].

Although previous studies [2,3,4,5,13,14] have reported the efficacy of ruxolitinib in treating steroid-resistant cGVHD with tolerable side effects, there are limited real-world data-based studies on the safety and efficacy of ruxolitinib salvage therapy in the peri-transplantation period of allo-HSCT in terms of GVHD risk [3,4,5,8,15]. Moreover, the correlation between the findings of these limited clinical studies and their applicability to daily clinical practice was poor. Therefore, this study was carried out to investigate the real-world outcomes of ruxolitinib as salvage therapy for steroid-resistant aGVHD, cGVHD, and oGVHD in patients undergoing allo-HSCT.

## 2. Materials and Methods

### 2.1. Study Design

This study was designed as a retrospective, observational multicenter study. The study protocol was approved by the Clinical Research Ethics Committee of Dr. Abdurrahman Yurtaslan Ankara Oncology Training and Research Hospital, University of Health Sciences, Ankara, Turkey (Approval Date: 23 June 2021, Approval Number: 2021-06/1254). The study was conducted per the ethical principles outlined in the Declaration of Helsinki. Written informed consent could not be obtained from the patients due to the study’s retrospective design and the data’s unanimity. 

### 2.2. Population and Sample

The study population consisted of 80 patients aged 18 years or older treated with ruxolitinib for Grade II or higher aGVHD, moderate-to-severe cGVHD, or oGVHD after allo-HSCT in nine medical centers across Turkey between January 2017 and December 2024. GVHD subtypes were diagnosed and graded as described in the literature [16,17,18]. Steroid resistance was defined based on the European Society for Blood and Marrow Transplantation (EBMT) criteria [12,17]. All patients in the study population met the study criteria and were included in the sample.

### 2.3. Data Collection

Patients’ demographic and clinical characteristics, including comorbidities, indications for allo-HSCT, allo-HSCT characteristics such as donor type, human leukocyte antigen (HLA) match status, prophylactic medications for GVHD, and conditioning regimens, were obtained from each medical center’s data-base and recorded on a standardized worksheet by independent reviewers at the respective medical center.

To enhance data consistency across participating centers, a standardized data collection template with predefined variable definitions was used. GVHD classification and response assessment were harmonized according to established criteria (including NIH consensus definitions for cGVHD). Submitted datasets were centrally reviewed for completeness and internal consistency, and discrepancies were clarified with the respective centers before final analysis.

### 2.4. Patient Groups

In addition to the cGVHD and aGvHD groups, we categorized cGVHD patients with aGvHD manifestations in the skin, liver, or gut into the oGVHD group [16,17]. We compared patients’ clinical characteristics, including the interval between allo-HSCT and development of GVHD, grade, organ involvement, and treatment modalities, between these groups. 

GVHD phenotypes were recorded longitudinally. Overlap GVHD was defined according to NIH/EBMT consensus terminology as the coexistence of diagnostic features of cGVHD together with manifestations characteristic of aGVHD. Because patients may evolve from acute to cGVHD over time, phenotype counts are not intended to be additive. For outcome reporting, denominators are explicitly stated for each analysis, and we clarify when subgroups overlap.

All enrolled patients had steroid-refractory GVHD at the time of ruxolitinib initiation. Steroid refractoriness was defined as progression after at least 3–5 days of high-dose corticosteroids or failure to achieve clinical response after ≥7 days of standard-dose steroid therapy, according to accepted clinical criteria.

### 2.5. Ruxolitinib Treatment

Ruxolitinib was initiated at 5 mg or 10 mg twice daily according to baseline hematologic parameters, infection risk, and physician discretion [8]. Patients with significant cytopenias at baseline were preferentially started at 5 mg twice daily, whereas those without hematologic contraindications generally received 10 mg twice daily. Dose adjustments during treatment were guided by hematologic tolerance, infectious complications, and clinical response [7]. In cases of grade ≥ 3 cytopenia or severe infection, dose reduction or temporary interruption was performed. Adverse events related to ruxolitinib may also lead to its discontinuation [19]. We recorded the duration of ruxolitinib treatment for each patient. 

### 2.6. Definitions

We defined overall survival (OS) as the interval between the initiation of ruxolitinib treatment and death and the last follow-up visit of patients [5,7] and calculated the 1-year, 2-year, and 5-year OS rates. 

We categorized treatment responses based on the National Institute of Health criteria [8]. Accordingly, complete resolution of all manifestations of GVHD was deemed to indicate complete response (CR), whereas a real clinical benefit indicated by an improvement in the assessment scores without complete resolution of all manifestations of GVHD was deemed to indicate partial response (PR). Patients without CR or PR were categorized into the no response (NR) category [8]. 

Response assessment was performed according to GVHD phenotype. For patients with aGVHD, treatment response was evaluated at day 28 following ruxolitinib initiation. For patients with cGVHD and NIH-defined oGVHD, response was assessed at approximately 3 months after treatment initiation, reflecting the slower response kinetics in chronic disease. Day-28 responses in cGVHD were recorded descriptively but were not considered definitive efficacy endpoints.

All participating centers documented organ involvement and treatment response according to NIH consensus criteria. For the purposes of this multicenter retrospective analysis, response categories (complete response, partial response, and no response) were harmonized using standardized NIH definitions. Data were extracted using structured case report forms, and when necessary, transplant physicians reviewed source documentation to ensure consistency in organ scoring and response categorization.

Infectious complications were retrospectively categorized as microbiologically documented infections, clinically documented infections without microbiological confirmation, or viral reactivation based on quantitative PCR monitoring. CMV reactivation was defined as detectable CMV DNAemia requiring antiviral intervention according to institutional thresholds. Invasive fungal infections were classified according to EORTC/MSG criteria when diagnostic criteria were met. Infection severity was graded according to CTCAE v5.0 where documentation permitted.

Institutional prophylaxis strategies across participating centers generally included antiviral prophylaxis (acyclovir or valacyclovir), *Pneumocystis jirovecii* prophylaxis (trimethoprim-sulfamethoxazole), and antifungal prophylaxis (fluconazole in standard-risk patients and posaconazole in selected high-risk cases). CMV surveillance was performed using serial quantitative PCR assays in accordance with allo-HSCT follow-up protocols.

### 2.7. Statistical Analysis

The results of the statistical analyses were expressed using descriptive statistics, i.e., mean ± standard deviation values in the case of continuous (numerical) variables determined to conform to the normal distribution, median with minimum and maximum values in the case of continuous variables determined not to conform to the normal distribution, and numbers and percentage values in the case of categorical variables. The normal distribution characteristics of numerical variables were analyzed using appropriate tests and visual tools, such as histograms and Q-Q (quantile-quantile) plots, depending on the sample size and data characteristics. Accordingly, the Shapiro–Wilk test was preferred in the case of small samples (n < 50), and Kolmogorov–Smirnov and Anderson-Darling tests were preferred in the case of large samples (n ≥ 50). 

Kaplan–Meier survival analysis and the log-rank test were used to assess any correlation between OS and various clinical factors based on the survival curves of all 80 patients. The clinical factors investigated within the scope of the study were selected based on their clinical importance and literature data, including their potential impact on survival. The log-rank test assessed whether the survival curves differed statistically significantly between these groups. In the Kaplan–Meier analysis, assumptions such as whether the data were censored and whether the survival times conformed to an independent distribution were considered, while the log-rank test assessed whether the survival curves differed statistically significantly between the study groups based on whether the expected and observed event counts were homogeneously distributed between them.

The primary endpoint was early overall response following ruxolitinib initiation, with phenotype-specific response timepoints applied according to GVHD subtype, calculated by dividing the sum of patients with CR or PR at the relevant date by the total number of patients [19], and the secondary outcome was the OS of all patients with any GVHD. 

In addition to early overall response, durability outcomes were evaluated descriptively. Duration of response (DoR) was defined as the time from first documented complete or partial response to loss of response, initiation of new systemic GVHD therapy, or death, whichever occurred first. Time to next systemic therapy was defined as the interval from ruxolitinib initiation to initiation of a subsequent systemic GVHD-directed treatment. Failure-free survival (FFS) was defined as survival without relapse of underlying disease, non-relapse mortality, or requirement for additional systemic GVHD therapy.

An exploratory multivariable Cox proportional hazards model was fitted to evaluate independent associations with OS. Given the limited number of events, the model was restricted to a small number of clinically prioritized covariates to minimize overfitting.

Statistical analyses were performed using Jamovi (Jamovi, version 2.3.28.0, 2023, retrieved from https://www.jamovi.org accessed 23 February 2026 and JASP (Jeffreys’ Amazing Statistics Program, version 0.19.0, 2024, retrieved from https://jasp-stats.org accessed 23 February 2026) software packages. Probability (*p*) statistics of ≤0.05 were deemed to indicate statistical significance.

## 3. Results

### 3.1. Overall Sample

The distribution of patients’ baseline demographic and clinical characteristics and allo-HSCT procedural features, including HLA match status (full HLA match, haploidentical, HLA mismatched HLA (9/10)) and regimen intensity [myeloablative conditioning (MAC)/reduced intensity (RIC)] is given in Table 1. The mean age of the sample, 60 males and 20 females, was 39.27 ± 13.30 years. Among 80 unique patients, 39 experienced aGVHD and 68 developed cGVHD during their disease course; 15 fulfilled NIH/EBMT criteria for oGVHD. These phenotype counts are not mutually exclusive. When categorized into mutually exclusive longitudinal patterns, 12 patients had aGVHD only, 41 had cGVHD only, 15 had oGVHD, and 12 showed sequential evolution from aGVHD to cGVHD without overlap features. The distribution of overlapping and mutually exclusive GVHD phenotypes is summarized in Table 2.

### 3.2. aGVHD Group

The distribution of clinical characteristics of patients with aGVHD (stage II/III/IV) is given in Table 3. 

A total of 39 patients developed aGVHD at a median duration of 55 (min. 17, max. 128) days after allo-HSCT. Of these patients, 15 (38.5%), 16 (41.0%), and 8 (20.5%) patients had grade II, III, and IV aGVHD, respectively. The most commonly involved organ was the skin (21 of 39 patients, 53.8%), followed by intestine (17 of 39 patients, 43.6%) and liver (14 of 39 patients, 35.9%). 27 (69.2%) patients had single-organ involvement, and 12 (30.7%) patients had multi-organ involvement. Only the liver, skin, and intestine were involved in 12 (30.8%), 10 (25.6%), and 5 (12.8%) patients, respectively. All patients received steroids as the first-line treatment modality. The ORR was 34 of 39 patients (87.2%), and the mortality rate was 16 of 39 patients (41.0%). 

Acute myeloid leukemia and acute lymphoblastic leukemia were the most common comorbidities seen in 30 (37.5%) and 28 (35.0%) patients, respectively. Matched-related donors (49 of 80, 61.3%) and matched-unrelated donors (14 of 80, 17.5%). In terms of the HLA-match status, 63 of 80 patients (78.8%) had a fully HLA-matched donor. As the GVHD prophylaxis, cyclosporin was the most frequently used medication (79 of 80, 98.8%), followed by steroids (n = 42, 52.5%), methotrexate (n = 36, 45.6%), and post-transplant cyclophosphamide (n = 30, 37.5%). 

### 3.3. cGVHD Group

The distribution of clinical characteristics of patients with cGVHD (moderate/severe) is given in Table 4. A total of 68 patients developed cGVHD at a median duration of 124.5 days after allo-HSCT. The median number of treatment modalities patients received prior to ruxolitinib treatment was 4 (min. 1, max. 8). Patients received ruxolitinib treatment for a median duration of 9 (min. 2.4, max. 68.2) months. The skin (54 of 68 patients (79.4%)) and liver (79.4%) were the most commonly affected organs. The ORR of the study group was 62 of 68 patients (91.2%), and the mortality rate was 21 of 68 patients (30.9%). 

### 3.4. oGVHD Group

The distribution of clinical characteristics of patients with oGVHD is given in Table 5. Skin (86.7%) and liver (86.7%) were the most frequently involved organs. The ORR of the study group was 13 of 15 patients (86.7%), and the mortality rate was 7 of 15 patients (46.7%). 

### 3.5. Ruxolitinib Treatment Outcomes

Ruxolitinib treatment outcomes, including treatment responses (ORR/no response), are shown in Table 6. The median duration of ruxolitinib treatment in the overall sample was 8 (min 2.4, max. 68.2) months. Ruxolitinib was initiated at 10 mg twice daily in 62 of 80 patients (77.5%), while 18 of 80 (22.5%) started at 5 mg twice daily, primarily due to baseline cytopenias. In terms of adverse effects, cytopenia occurred in 23 of 80 patients (28.7%), anemia in 18 of 80 patients (22.5%), neutropenia in 19 of 80 patients (23.8%), and thrombocytopenia in 12 of 80 patients (15.0%). A total of 40 (50%) patients developed an infection associated with ruxolitinib. Of these 40 patients, 20 infected patients (50.0%) developed respiratory infections, and 10 (25%) developed gastrointestinal infections. A total of 24 of 80 (30%) patients had virus reactivation, of which 20 patients (25%) had cytomegalovirus (CMV) reactivation and 4 (5%) had herpes simplex virus (HSV) reactivation. Infectious complications occurred in 40 of 80 patients (50%) during ruxolitinib treatment. Viral reactivation was observed in 24 patients (30%), most commonly CMV DNAemia in 20 patients (25%). Non-CMV infections were documented in 40 patients, predominantly bacterial in origin, while fungal infections were infrequent and rarely microbiologically confirmed. Infections accounted for 15 of 26 deaths (58.3%), underscoring the substantial infectious burden in this heavily immunosuppressed population. The majority of infectious events occurred within the first 6 months following ruxolitinib initiation.

Ruxolitinib was discontinued in 51 of 80 (63.7%) patients, primarily due to loss of treatment response (15 of 51, 29.4%) and mortality (11 of 51, 21.6%). Following ruxolitinib treatment, PR and CR were achieved in 35 (43.8%) and 37 (46.3%) patients, respectively, while 8 (10%) patients had NR. Accordingly, the ORR of the sample was 90%. Five (6.2%) patients relapsed.

Failure-free survival analysis demonstrated a median FFS of 16.8 months (range 1.8–62.3). The 1-year failure-free survival rate was 58.8%. This reflects survival without relapse of the underlying disease, non-relapse mortality, or documented loss of response. Due to the retrospective design and absence of systematically recorded response onset dates across centers, formal duration of response calculation was limited; however, time to documented response loss among responders was incorporated into FFS estimation. 

**Figure 1:**
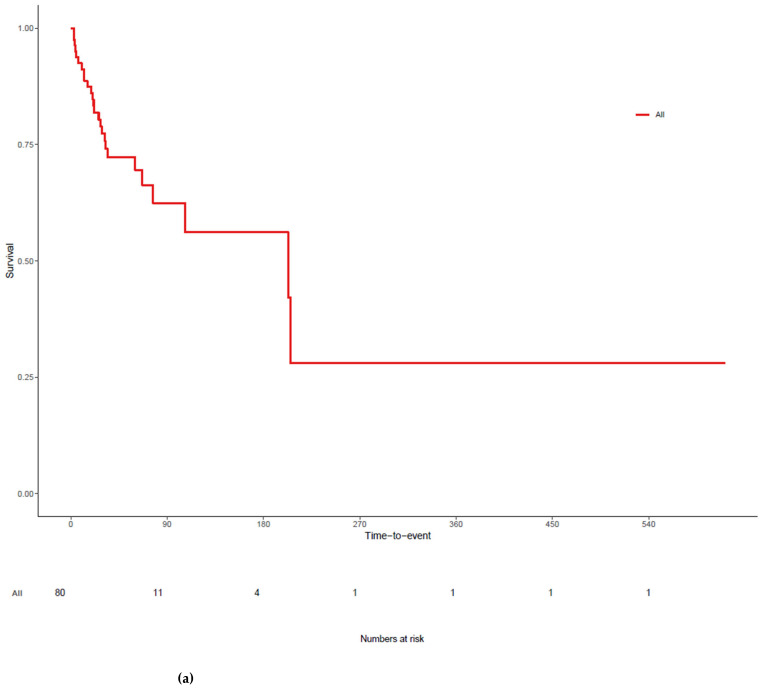

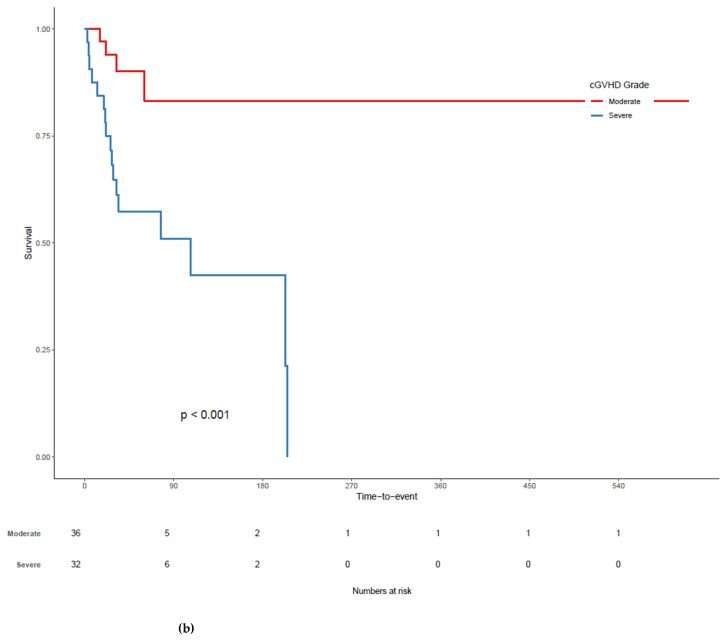

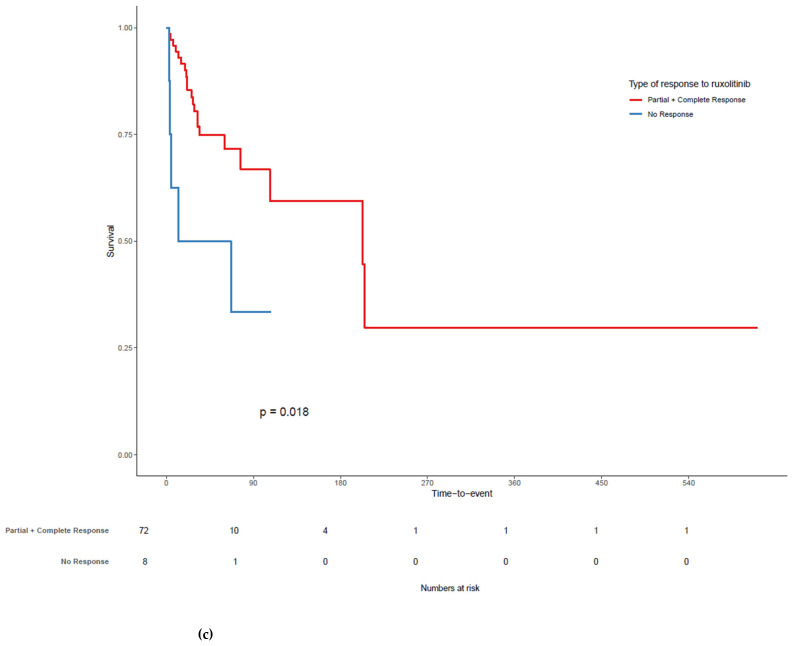
(**a**) The overall survival of all patients treated with ruxolitinib for graft-versus-host disease; (**b**) Kaplan–Meier survival analysis with Log-Rank test of patients with moderate and severe chronic graft-versus-host disease; (**c**) Kaplan–Meier survival analysis with Log-Rank test of patients with and without ruxolitinib response; Numbers at risk are shown below the x-axis. Survival percentages are based on Kaplan–Meier estimation for all figures.

The mortality rate of the sample was 26 of 80 patients (32.5%). Infections accounted for 15 of 26 deaths (58.3%, Table 5). Overall survival rate at last follow-up was 67.5%, reflecting the proportion of patients alive at data cutoff rather than survival at a predefined timepoint. The median follow-up duration was 32.3 months, calculated using the reverse Kaplan–Meier method. The median OS was 34.5 (min. 2.7, max. 61.1) months (Figure 1a), whereas the 1-year, 2-year, and 5-year OS rates were 91.3%, 82.5%, and 25.0%, respectively. Kaplan–Meier survival analysis and log-rank test indicated that severe cGVHD (*p* < 0.001) (Figure 1b) and lack of response to ruxolitinib treatment (*p* = 0.018) (Figure 1c) were significantly associated with reduced OS, whereas aGVHD grade (*p* = 0.221), HLA match status (*p* = 0.573), and regimen intensity (*p* = 0.873) were not significantly correlated with OS. In an exploratory multivariable Cox model adjusted for severe cGVHD, response to ruxolitinib, and age, severe cGVHD remained independently associated with inferior OS (HR 2.63, 95% CI 1.14–6.10, *p* = 0.024). Lack of response to ruxolitinib was associated with increased hazard with borderline significance (HR 2.64, 95% CI 0.94–7.42, *p* = 0.066), whereas age was not significant (HR 0.98 per year, 95% CI 0.95–1.02, *p* = 0.319). Detailed regression coefficients and model parameters are presented in Supplementary Table S1.

## 4. Discussion

The study findings revealed a high ORR (90%) for ruxolitinib treatment in patients with allo-HSCT and indicated a manageable safety profile, supporting the use of ruxolitinib in a setting with limited treatment options. High 1-year and 2-year OS rates further underscored the potential of ruxolitinib as a viable treatment option for GVHD.

A systematic review and meta-analysis study reported a wide range of ORR to ruxolitinib treatment, ranging from 45 to 82% in patients with steroid-resistant aGVHD and 75 to 100% in those with cGVHD [4,20]. A multicenter study conducted with 95 patients who received ruxolitinib as salvage therapy for steroid-resistant GVHD reported the ORR as 81.5% and 85.4% and 6-month OS rate as 79% and 97.4% in patients with aGVHD and cGVHD, respectively [6]. In comparison, we calculated the 1-year and 2-year OS rates for our overall study group as 91.3% and 82.5%, respectively. Some studies have reported lower CR or PR rates in patients with steroid-resistant aGVHD and 1-year OS rates as low as 41.5%, suggesting that factors such as aGVHD grade, treatment sequencing, and patient performance status may significantly influence the outcomes of ruxolitinib treatment [5]. Prospective studies where the heterogeneity caused by patient populations is controlled are needed to definitively assess the efficacy and safety of ruxolitinib.

Several studies have explored the efficacy of ruxolitinib as salvage therapy for GVHD. In one of these studies evaluating the efficacy of ruxolitinib for multidrug-resistant GVHD, Zhao et al. [4] found the ORR as 60.0% for aGVHD and 89.5% for cGVHD. Although hematologic and infectious toxicities were common, ranging from 36.8% to 73.3%, they concluded that these results suggest that ruxolitinib is a promising salvage therapy for multidrug-resistant GVHD. Similarly, Javed et al. [3] reported an ORR of 85% in a cohort of seven aGVHD and 13 cGVHD patients treated for steroid-resistant GVHD, with adverse events occurring in 80% of patients. Consistent with these findings, the ORR was 90% in our sample, 91.3% in the cGVHD group, and 86.7% in the oGVHD group. Although infectious and hematological adverse effects were common in our sample, as in those of other studies, these adverse effects did not lead to treatment discontinuation [3,4,8]. These findings collectively suggest that ruxolitinib may be a valuable option for treating GVHD in patients undergoing allo-HSCT. 

In an exploratory multivariable Cox regression analysis adjusting for severe cGVHD status, response to ruxolitinib, and age, severe cGVHD remained independently associated with inferior overall survival. Lack of response to ruxolitinib was associated with increased hazard with borderline statistical significance, whereas age was not independently associated with mortality. These findings suggest that disease severity at the time of ruxolitinib initiation is a key determinant of survival, even after limited adjustment for confounding factors.

Among the important side effects associated with ruxolitinib, infectious complications were observed in nearly half of the patients in our sample, consistent with previous reports [4]. We found the safety profile of ruxolitinib to be more favorable compared to other studies in the literature in terms of CMV reactivation, which occurred in 25% of our sample [6]. Overall, the safety profile of ruxolitinib is superior to other therapeutic agents in patients with allo-HSCT [4,7,19,20,21]. The substantial infectious burden observed in our cohort aligns with the profound immunosuppression associated with steroid-refractory GVHD and the additional impact of JAK inhibition. The updated ECIL 2025 guidelines on primary antifungal prophylaxis in hematological malignancies emphasize the importance of mold-active prophylaxis in high-risk allo-HSCT recipients receiving multiple immunosuppressive agents. Although antifungal prophylaxis was routinely administered in our centers, variability in agent selection and duration may have influenced infection patterns. Prospective studies with standardized surveillance and prophylaxis strategies are warranted.

The relatively lower non-response and CMV reactivation rates observed in our cohort compared with some previously published studies may be attributable to several factors. Differences in patient selection, timing of ruxolitinib initiation, and prior lines of therapy may have influenced response rates. Additionally, structured antiviral prophylaxis and routine PCR-based CMV surveillance across centers may have contributed to lower clinically significant CMV reactivation. Variability in definitions and reporting thresholds between studies, as well as the retrospective nature of our analysis, may also partially explain these differences.

Unresponsiveness or intolerance to ruxolitinib is another critical issue noted in prior studies. The REACH2 (Research to Assess Spiration Valve System Safety and Effectiveness for the Treatment of Severe Emphysema in China) trial reported NR as 38% on day 28 and 60% on day 56 [22], whereas Zeiser et al. reported NR as 14.6% [6]. The median incidence of ruxolitinib resistance in adult GVHD patients was reported as 21% based on real-world data [23,24], and NR was found to correlate with reduced OS [5,8]. In comparison, the NR rate in our sample was 8 of 80 patients (10.0%). We also found a significant correlation between NR and reduced OS. These findings collectively underscore the importance of early assessment in patients with NR to adjust treatment strategies promptly.

While our study provided valuable insights into the efficacy and safety of ruxolitinib as salvage therapy for GVHD in a real-world setting, several limitations should be considered when interpreting the results. First, its retrospective design inherently limited the ability to establish causal relationships between ruxolitinib treatment and outcomes. Secondly, the absence of a control group or comparator arm limited the ability to directly compare ruxolitinib with other available therapies or standard treatment protocols. Thirdly, the fact that the study population consisted of patients with varying types and severities of GVHD (acute, chronic, and overlap), who had different donor types and conditioning regimens, and received various treatments prior to ruxolitinib treatment might have introduced confounding factors that could potentially influence response rates and survival outcomes, limiting the generalizability of the results to specific GVHD subgroups. In this regard, even though we analyzed multiple clinical variables, our study does not fully account for all potential confounders, including baseline GVHD severity, Eastern Cooperative Oncology Group (ECOG) performance status, comorbidities, or concurrent treatments that may impact response and survival. Although infections accounted for a substantial proportion of deaths, formal competing-risk modeling distinguishing non-relapse mortality from relapse-related mortality was not performed. Given the limited number of cause-specific events and the retrospective design, competing-risk regression would have resulted in unstable estimates. Therefore, causes of death were presented descriptively. Future larger studies with adequate event numbers should incorporate competing-risk methodology to better delineate mortality patterns in this population. The relatively small number of events limited the number of covariates that could be included in the multivariable model. To minimize overfitting, only a small number of clinically prioritized variables were entered into the Cox regression model. Consequently, residual confounding cannot be excluded, and findings should be interpreted as exploratory rather than definitive evidence of independent causality. Detailed microbiological documentation was not uniformly available across centers, and infection grading was limited by retrospective data collection. Therefore, the reported infectious complications should be interpreted within the constraints of real-world documentation variability.

In conclusion, although our study adds to the growing body of evidence supporting the use of ruxolitinib in the treatment of GVHD, these findings would be better viewed as a stepping stone toward more comprehensive research efforts. Future prospective, randomized, controlled, long-term, follow-up studies addressing the limitations of our study will be crucial in establishing standardized protocols for the use of ruxolitinib in GVHD; improving associated patient outcomes; considering the impact of organ-specific involvement, treatment sequencing, and combination therapies; and understanding ruxolitinib’s safety profile, particularly in terms of infectious complications and OS. In this multicenter retrospective cohort, ruxolitinib was associated with high response rates and acceptable toxicity in heavily pretreated GVHD patients. However, given the observational design and limited event number, these findings should be interpreted as exploratory associations rather than definitive evidence of causal efficacy.

## Figures and Tables

**Table 1:** Baseline demographic and clinical characteristics of the patients (n = 80).

Variables		Value
Age (year)		40.63 ± 13.23
Sex ^‡^	Female	20 (25.0)
	Male	60 (75.0)
Comorbidities ^‡^	Hypertension	10 (12.5)
	Diabetes mellitus	5 (6.2)
	Others	9 (11.2)
Diagnosis ^‡^	Acute myeloid leukemia	30 (37.5)
	Acute lymphoblastic leukemia	28 (35.0)
	Hodgkin’s lymphoma	3 (3.8)
	Chronic myelogenous leukemia	4 (5.0)
	Chronic myelomonocytic leukemia	3 (3.8)
	Myelodysplastic syndrome	3 (3.8)
	Others	9 (11.2)
Donor type ^‡^	Matched related donor	49 (61.3)
	Matched unrelated donor	14 (17.5)
	Haplo-identical donor	5 (6.2)
	HLA-mismatched (related/unrelated) (9/10)	12 (15.0)
HLA match status ^‡^	Full HLA match	63 (78.8)
	Haplo-identical donor	5 (6.2)
	HLA-mismatched (HLA 9/10)	12 (15.0)
GVHD prophylaxis ^‡^	Cyclosporine	79 (98.8)
	Steroids	42 (52.5)
	Methotrexate	36 (45.6)
	Posttransplant cyclophosphamide	30 (37.5)
	Anti-T-lymphocyte globulin	20 (25.0)
	Mycophenolate mofetil	8 (10.0)
	Tacrolimus	2 (2.5)
	Sirolimus	1 (1.2)
Conditioning regimens ^‡^	Busulfan-based	60 (75.0)
	Total body irradiation-based	12 (15.0)
	Treosulfan-based	2 (2.5)
	Others	6 (7.5)
Regimen intensity ^‡^	Myeloablative conditioning	75 (87.5)
	Reduced intensity	5 (12.5)
GVHD types ^‡^	Acute	39 (48.8)
	Chronic	68 (85.0)
	Overlapping	15 (18.8)

HLA: human leukocyte antigen, GVHD: graft-versus-host disease; ^‡^: n (%). Percentages are calculated using the relevant subgroup denominator unless otherwise specified. GVHD phenotype counts reflect longitudinal occurrence and are not mutually exclusive; therefore, totals may exceed 80. Overlap GVHD is a subset of patients with concurrent acute and chronic features per NIH/EBMT criteria.

**Table 2:** Distribution of GVHD phenotypes and longitudinal patterns in the total cohort (n = 80).

Variables		n
Total cohort (unique patients)		80
Phenotypes during disease course (not mutually exclusive)	aGVHD ever	39
	cGVHD ever	68
	NIH-overlap	15
Mutually exclusive longitudinal pattern (sums to 80)	aGVHD only	12
	cGVHD only	41
	NIH-overlap GVHD	15
	Sequential evolution from aGVHD to cGVHD without overlap features	12

**Table 3:** Clinical characteristics of patients with aGVHD (n = 39; longitudinal phenotype).

Variables		Value
Interval between allo-HSCT and aGVHD (day) ^§^		55.0 [17.0–128.0]
Number of treatment lines prior to ruxolitinib ^§^		4.0 [2.0–8.0]
Ruxolitinib duration (month) ^§^		7.5 [2.4–68.2]
Grade ^‡^	Grade II	15 (38.5)
	Grade III	16 (41.0)
	Grade IV	8 (20.5)
Organ involvement ^‡^	Skin	21 (53.8)
	Intestine	17 (43.6)
	Liver	15 (38.5)
Involved organ modalities ^‡^	Skin	10 (25.6)
	Liver	12 (30.8)
	Intestine	5 (12.8)
	Skin+intestine	9 (23.1)
	Skin+intestine+liver	2 (5.1)
	Intestine+liver	1 (2.6)
Number of involved organs ^‡^	1	27 (69.2)
	2	10 (25.6)
	3	2 (5.1)
Treatment modalities for aGVHD ^‡^	Steroids	39 (100.0)
	Cyclosporine-A	34 (87.2)
	Mycophenolate mofetil	29 (74.4)
	Photopheresis	18 (46.2)
	Methotrexate	7 (17.9)
	Mesenchymal cell	6 (15.4)
	Tacrolimus	5 (12.8)
	Cyclosporine	2 (5.1)
	Anti-T-lymphocyte globulin	2 (5.1)
Type of response to ruxolitinib ^‡^	Partial response	17 (43.6)
	Complete response	17 (43.6)
	No response	5 (12.8)
	Overall response rate	34 (87.1)
Outcome ^‡^	Survived	23 (59.0)
	Non-survived	16 (41.0)

Allo-HSCT: allogeneic hematopoietic stem cell transplantation, aGVHD: acute graft-versus-host disease; ^‡^: n (%), ^§^: Median [min-max]. Percentages are calculated using the relevant subgroup denominator unless otherwise specified.

**Table 4:** Clinical characteristics of patients with cGVHD (n = 68; longitudinal phenotype).

Variables		Value
Interval between allo-HSCT and cGVHD (day) ^§^		124.5 [17.0–996.0]
Number of treatment lines prior to ruxolitinib ^§^		4.0 [1.0–8.0]
Ruxolitinib duration (month) ^§^		9.0 [2.4–68.2]
Grade ^‡^	Moderate	36 (52.9)
	Severe	32 (47.1)
Organ involvement ^‡^	Skin	55 (80.9)
	Eye	21 (30.9)
	Mouth	35 (51.5)
	Lung	13 (19.1)
	Liver	54 (79.4)
	Intestine	15 (22.1)
	Joints	11 (16.1)
	Others (Kidney, genital organs, pericardial effusion)	4 (5.9)
Number of involved organs ^‡^	1	19 (27.9)
	2	3 (4.4)
	3	25 (36.8)
	≥4	21 (30.9)
Treatment modalities for cGVHD ^‡^	Steroids	67 (98.5)
	Cyclosporine-A	52 (76.5)
	Mycophenolate mofetil	40 (58.8)
	Photopheresis	39 (57.4)
	Ibrutinib	14 (20.6)
	Methotrexate	13 (19.1)
	Rituximab	13 (19.1)
	Tacrolimus	12 (17.6)
	Imatinib	10 (14.7)
	Bortezomib	5 (7.4)
	Cyclophosphamide	3 (4.4)
	Sirolimus	3 (4.4)
	Mesenchymal stromal cells	3 (4.4)
	Infliximab	1 (1.5)
Type of response to ruxolitinib ^‡^	Partial response	32 (47.1)
	Complete response	31 (45.6)
	No response	5 (7.4)
	Overall response rate	63 (91.3)
Outcome ^‡^	Survived	47 (69.1)
	Non-survived	21 (30.9)

Allo-HSCT: allogeneic hematopoietic stem cell transplantation, cGVHD: chronic graft-versus-host disease; ^‡^: n (%), ^§^: Median [min-max]. Percentages are calculated using the relevant subgroup denominator unless otherwise specified.

**Table 5:** Clinical characteristics of patients with NIH-overlap GVHD (n = 15; subset of patients with both aGVHD and cGVHD).

Variables		Value
Interval between allo-HSCT and oGVHD (day) ^§^		50.0 [17.0–125.0]
Number of treatment lines prior to ruxolitinib ^§^		4.0 [2.0–5.0]
Ruxolitinib duration (month) ^§^		7.0 [2.4–54.8]
Organ involvement ^‡^	Skin	13 (86.7)
	Eye	6 (40.0)
	Mouth	8 (53.3)
	Lung	4 (26.7)
	Liver	13 (86.7)
	Intestine	1 (6.7)
	Others (Kidney, genital organs, pericardial effusion)	1 (6.7)
Number of involved organs ^‡^	1	3 (20.0)
	2	2 (13.3)
	3	4 (26.7)
	≥4	6 (40.0)
Treatment modalities for oGVHD ^‡^	Steroids	15 (100.0)
	Mycophenolate mofetil	12 (80.0)
	Cyclosporine-A	11 (73.3)
	Photopheresis	11 (73.3)
	Tacrolimus	5 (33.3)
	Ibrutinib	5 (33.3)
	Rituximab	3 (20.0)
	Methotrexate	2 (13.3)
	Imatinib	2 (13.3)
	Cyclophosphamide	1 (6.7)
	Mesenchymal stromal cells	1 (6.7)
Type of response to ruxolitinib ^‡^	Partial response	7 (46.7)
	Complete response	6 (40.0)
	No response	2 (13.3)
	Overall response rate	13 (86.7)
Outcome ^‡^	Survived	8 (53.3)
	Non-survived	7 (46.7)

Allo-HSCT: allogeneic hematopoietic stem cell transplantation, oGVHD: overlapping graft-versus-host disease; ^‡^: n (%), ^§^: Median [min-max]. Patients in the overlap group are included within the cGVHD and aGVHD phenotype counts. Percentages are calculated using the relevant subgroup denominator unless otherwise specified.

**Table 6:** Use of ruxolitinib in the study group (n = 80).

Variables			Values
Number of treatment lines prior to ruxolitinib ^§^			4.0 [1.0–8.0]
Roxolitinib duration (month) ^§^			8.0 [2.4–68.2]
Initiation dose ^‡^		5 mg once a day	7 (8.8)
		5 mg twice a day	7 (8.8)
		10 mg once a day	2 (2.5)
		10 mg twice a day	62 (77.5)
		Others	2 (2.5)
Adverse effects ^‡^	Cytopenia		23 (28.7)
		Anemia	18 (22.5)
		Neutropenia	19 (23.8)
		Thrombocytopenia	12 (15.0)
	Infections		40 (50.0)
		Respiratory	20 (50.0)
		Gastrointestinal	10 (25.0)
		Urinary	3 (7.5)
		Soft tissue	3 (7.5)
		Others	4 (10.0)
	Virus reactivation		24 (30.0)
		CMV reactivations ^‡^	20 (25.0)
		HSV reactivations ^‡^	4 (5.0)
	Skin eruption		2 (2.5)
	Nausea vomiting		5 (6.2)
Ruxolitinib withdrawal ^‡^			51 (63.7)
	Reasons ^‡^	Loss of clinical response under ruxolitinib	15 (29.4)
		Mortality	11 (21.6)
		Clinical response	10 (19.6)
		Adverse effects	5 (9.8)
		Relapsing disease under ruxolitinib	5 (9.8)
		Lack of medication	3 (5.9)
		Secondary cancer development	2 (3.9)
Type of response to ruxolitinib ^‡^		Partial response	35 (43.8)
		Complete response	37 (46.3)
		No response	8 (10.0)
		Overall response rate	72 (90.0)
Outcome ^‡^		Survived	54 (67.5)
		Non-survived	26 (32.5)
Relapsed disease under ruxolitinib treatment ^‡^			5 (6.3)
Reason for mortality ^‡^		Infections	14 (58.3)
		Disease-related	6 (25.0)
		Others	4 (16.7)
Overall survival (month) ^§^			34.5 [2.7–61.1]
1-year survival ^‡^			73 (91.3)
2-year survival ^‡^			66 (82.5)
5-year survival ^‡^			20 (25.0)

CMC: Cytomegalovirus, HSV: Herpes simplex virus; ^‡^: n (%), ^§^: Median [min–max]. Overall response rate (ORR) for the entire cohort is calculated using unique patients (n = 80). Subgroup ORRs are descriptive and reported within each phenotype-defined subgroup; overlap GVHD represents a subset and may therefore appear within more than one phenotype count. Percentages are calculated using the relevant subgroup denominator unless otherwise specified.

## Data Availability

The data will not be shared because permission was not obtained for sharing them during the ethical committee approval process for the multicenter study.

## Supplementary Materials

The following supporting information can be downloaded at: https://www.mdpi.com/article/10.3390/jcm15052088/s1, Supplementary Table S1. Multivariable Cox proportional hazards model for overall survival.
