# Supplementary material for: Real-World Outcomes of Ruxolitinib as Salvage Therapy in Steroid-Refractory Acute and Chronic Graft-Versus-Host Disease: A Multicenter Retrospective Observational Study from Turkey
Source: J Clin Med. 2026 Mar 9;15(5):2088. doi: 10.3390/jcm15052088 (PMC12986514; doi:10.3390/jcm15052088)
Supplement: Supplementary file 1 [file jcm-15-02088-s001.zip › jcm-4158547-supplementary.pdf]

Supplementary Table S1. Multivariable Cox proportional hazards model for overall survival

Variable	Hazard Ratio (HR)	95% Confidence Interval	p-value
Severe cGVHD (vs moderate)	2.63	1.14–6.10	0.024
No response to ruxolitinib (vs partial/complete response)	2.64	0.94–7.42	0.066
Age at ruxolitinib initiation (per year)	0.98	0.95–1.02	0.319

*Model adjusted for severe cGVHD status, response to ruxolitinib, and age at treatment initiation. The number of events was 26. Variables were limited to minimize overfitting based on event-per-variable considerations.
